# Refractory Intracranial Hypertension Due to Fentanyl Administration Following Closed Head Injury

**DOI:** 10.3389/fneur.2013.00003

**Published:** 2013-01-28

**Authors:** Sara E. Hocker, Jeremy Fogelson, Alejandro A. Rabinstein

**Affiliations:** ^1^Division of Critical Care Neurology, Mayo ClinicRochester, MN, USA; ^2^Department of Neurological Surgery, Mayo ClinicRochester, MN, USA

**Keywords:** closed head injury, intracranial hypertension, opioids, fentanyl, morphine, traumatic brain injury

## Abstract

**Background:** Although the effects of opioids on intracranial pressure (ICP) have long been a subject of controversy, they are frequently administered to patients with severe head trauma. We present a patient with an uncommon paradoxical response to opioids.

**Case Report:** A patient with refractory intracranial hypertension after closed head injury was managed with standard medical therapy with only transient decreases in the ICP. Only after discontinuation of opiates did the ICP become manageable without metabolic suppression and rescue osmotic therapy, implicating opiates as the etiology of refractory intracranial hypertension in this patient.

**Conclusion:** Clinicians should consider opioids as a contributing factor in malignant intracranial hypertension when findings on neuroimaging do not explain persistent and refractory intracranial hypertension.

## Introduction

Although opioids frequently are administered to patients with severe head trauma, the effects of such drugs on intracranial pressure (ICP) are controversial (Marx et al., 1989; Cuillerier et al., 1990; Weinstabl et al., 1991; Markovitz et al., 1992). Fentanyl and other opiates may cause a direct increase in ICP in certain patients although the mechanism and clinical significance of this effect is currently unknown (Sperry et al., 1992; Tobias, 1994; de Nadal et al., 2000). Despite these isolated reports, widespread experience supports the safety of fentanyl for patients with elevated ICP in head trauma.

## Case Report

Our patient was a 19-year-old woman who was the restrained driver in a roll-over accident at highway speed. Her Glasgow coma scale was 4 with extensor posturing. She was sedated, paralyzed, intubated, and transported to our hospital as a level 1 trauma. Her medical history was unremarkable.

**Figure 1:**
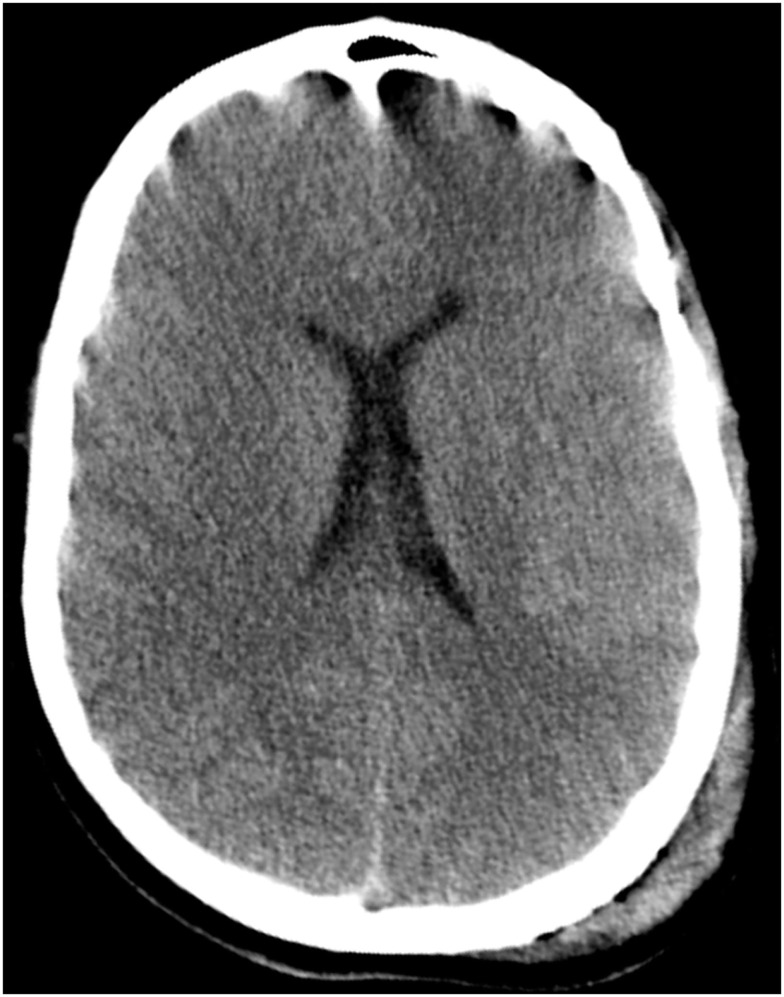
**CT scan of the head on hospital day 1 demonstrates poor gray-white junction differentiation suggestive of diffuse cerebral edema**. There are scattered areas of subarachnoid hemorrhage in multiple frontal and parietal sulci as well as scattered areas of hyperattenuation at the gray-white junction in the frontal and parietal lobes which may be related to contusions or diffuse axonal injury. Also seen is a large left parietal scalp hematoma.

CT imaging of the head revealed multiple punctate hemorrhagic lesions, early effacement of the gray-white junction and diffuse traumatic subarachnoid hemorrhage (Figure 1).

**Figure 2:**
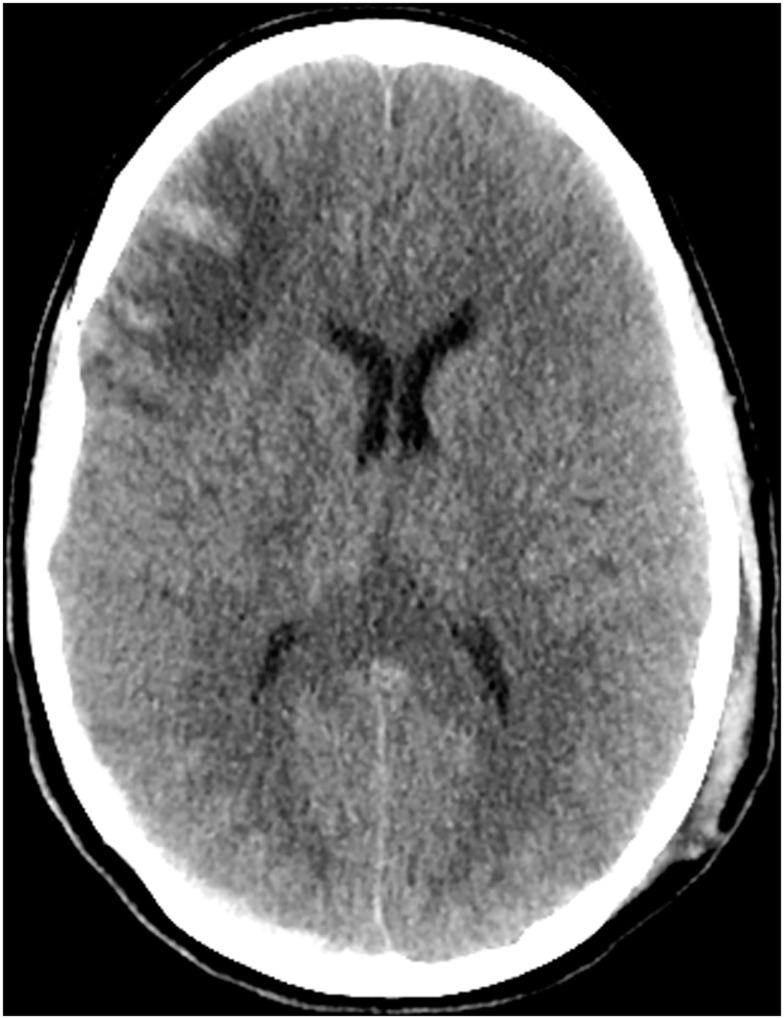
**CT scan of the head on hospital day 11 demonstrates expected evolution of the previously noted cortical contusions, subdural hematomas, and changes of diffuse axonal injury**. No new hemorrhage, mass effect, hydrocephalus, or CT radiographic evidence of acute ischemic injury. Stable diffuse cerebral swelling with partial effacement of the suprasellar cistern.

**Figure 3:**
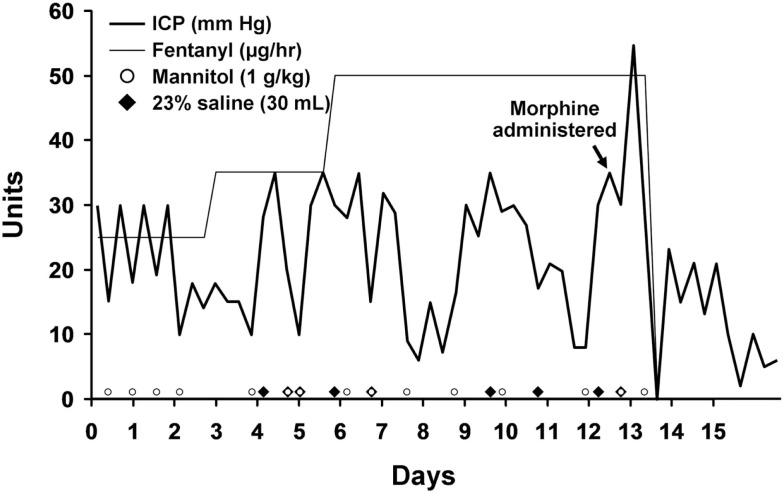
**Intracranial pressure is plotted over time**. The timing of morphine and fentanyl administration are shown. Rescue doses of hyperosmolar therapy are plotted at the bottom of the graph.

An intraparenchymal ICP monitor was placed with initial readings of 23–25 mmHg. Supportive care was instituted with midline placement of the head and elevation of the head to 30°, sedation with propofol and analgesia with fentanyl. She required bolus doses of 20% mannitol for ICP surges with physical stimulation. On hospital day 3 she developed refractory intracranial hypertension and fever which persisted through hospital day 12 despite aggressive medical management. She was very sensitive to any physical stimulation, particularly changes in position or tracheal suctioning which resulted in ICP spikes. Osmotic therapy including alternating boluses of 20% mannitol and 23% saline were used to treat ICP surges. Maintenance fluids were changed to 3% saline solution in order to induce mild hypernatremia and decrease the need for rescue osmotic therapy. We induced mild hypothermia (35°C) using the Arctic Sun device (Bard, Inc., Atlanta, GA, USA) as an adjuvant to osmotic therapy with pharmacological neuromuscular paralysis to control the shivering response. Several attempts at reducing the degree of metabolic suppression by slowly increasing the core body temperature and lightening the degree of neuromuscular paralysis and propofol requirement failed. She consistently required a temperature under 35°C and a dose of Propofol greater than 40 mcg/kg/min to maintain control of her ICP. A repeat CT scan on hospital day 11 (Figure 2) did not show significant cerebral edema to explain the persistent and refractory intracranial hypertension. It was noted that despite adequate hydration and apparent intravascular euvolemia, she was consistently tachycardic with intermittent periods of hypertension. This raised the possibility of sympathetic hyperactivity for which she was started on propranolol with only marginal decrease in the heart rate. On hospital day 12 a bolus of 4 mg of morphine was administered following a rise in ICP to 30 mmHg in an effort to determine if the elevation in ICP was due to a sympathetic surge. Immediately following the administration of morphine the ICP rose steadily from a baseline of 30 mmHg up to 55 mmHg over 3 min without any appreciable change in heart rate or blood pressure. The transient but significant rise in ICP was easily managed with hyperventilation and administration of 1 g/kg of 20% mannitol. Following this unexpected surge in ICP after administration of morphine, the fentanyl infusion she had been receiving since hospital day 1 was discontinued with an ensuing decline in ICP over the next 24 h which reached single digits for the first time since monitoring was started. No new interventions were introduced during this period. Parallel with the normalization of ICP was a resolution of her persistent sinus tachycardia and intermittent arterial hypertension. Permitting transient increases of ICPs up to 30 mmHg after stimulation, we were able to rapidly resume normothermia (36.5°C), discontinue neuromuscular paralysis, and transition from propofol to a low dose of midazolam without the need for rescue doses of hyperosmolar therapy (Figure 3). Before discontinuation of the ICP monitoring, her ICPs had steadily settled below 15 mmHg.

## Discussion

Morphine and fentanyl are the most commonly used opioids for analgesia in patients suffering head trauma (Shapiro et al., 1995). Studies in humans have shown varying effects of opioid administration on ICP with some patients developing substantial transient rises after bolus dosing (Moss et al., 1978; Marx et al., 1989; Cuillerier et al., 1990; Weinstabl et al., 1991; Markovitz et al., 1992; Sperry et al., 1992; Albane‘se et al., 1999). Most of these studies find a parallel decrease in mean arterial blood pressure (MAP). The decrease in MAP has been hypothesized to be the mechanism of increased ICP observed after administration of opioids in patients with reduced intracranial compliance and intact autoregulation through a pathway of vasodilation and increased blood volume (Werner et al., 1995). Our patient did not have a concomitant decrease in MAP making this mechanism improbable.

Another possible mechanism is a direct vasodilatory effect associated with opioid administration (Wahl, 1985; Thorogood and Armstead, 1995). The fact that she was so sensitive to hypothermia and propofol administration, interventions which reduce cerebral metabolism and blood volume, also support a theory of increased blood volume due to vasodilatation. Opioid-induced rigidity has been suggested as a possible mechanism of increased ICP following opiate administration due to increasing cerebral metabolic rate for oxygen (CMRo2), cerebral blood flow (CBF), and/or cerebral blood volume (Benthuysen et al., 1988). This is unlikely, however, to have been a significant contributing factor in our patient because she was under nearly complete neuromuscular blockade during the period of refractory intracranial hypertension.

An alternative mechanism which could induce increased ICP indirectly is opioid-induced histamine release by a vasodilatory effect and resultant increased CBF. Our patient consistently responded to osmotic agents but always briefly. This was likely due to a rheologic reduction in cerebral blood volume rather than to osmotic diuresis as she did not have evidence of significant cerebral edema on imaging. While this may explain the sudden increase in ICP following morphine administration in our patient, it does not fully explain the reduction in ICP after discontinuation of fentanyl infusion as fentanyl does not typically trigger histamine release (Flacke et al., 1987). This mechanism is also made unlikely by the lack of significant hemodynamic change associated with the rise in ICP. Non-opiate related reasons such as the natural history of the disease may exist to explain the phenomenon we observed.

## Conclusion

Although the mechanism refractory intracranial hypertension in our patient is unclear, the sudden surge in ICP following morphine administration in addition to the resolution of refractory intracranial hypertension following discontinuation of fentanyl infusion strongly argues for a causal relationship. The mechanism of the increased ICP in our patient is unclear, however, it is reasonable to assume that it could be related to intrinsic properties of opioids such as direct cerebral vasodilation combined with some predisposition in our patient. Despite widespread use of opiates in patients with head injury, we encourage the clinician to consider opiates as a possible cause for refractory intracranial hypertension in patients at risk for decreased intracranial compliance, particularly if a sufficient explanation for persistent intracranial hypertension is lacking.

## Conflict of Interest Statement

The authors declare that the research was conducted in the absence of any commercial or financial relationships that could be construed as a potential conflict of interest.
